# Quantitative Evaluation System of Upper Limb Motor Function of Stroke Patients Based on Desktop Rehabilitation Robot

**DOI:** 10.3390/s22031170

**Published:** 2022-02-03

**Authors:** Mingliang Zhang, Jing Chen, Zongquan Ling, Bochao Zhang, Yanxin Yan, Daxi Xiong, Liquan Guo

**Affiliations:** 1 School of Biomedical Engineering (Suzhou), Division of Life Sciences and Medicine, University of Science and Technology of China, Suzhou 215163, China; zmlxjq@mail.ustc.edu.cn (M.Z.); sle1008@mail.ustc.edu.cn (J.C.); lzq597@mail.ustc.edu.cn (Z.L.); zbc2020@mail.ustc.edu.cn (B.Z.); xiongdx@sibet.ac.cn (D.X.); 2 Suzhou Institute of Biomedical Engineering and Technology, Chinese Academy of Sciences, Suzhou 215163, China; 3 Center for Excellence in Brain Science & Intelligence Technology, Institute of Neuroscience, Chinese Academy of Sciences, Shanghai 200031, China; yanxin.yan@icpbr.ac.cn

**Keywords:** stroke, rehabilitation training, movement evaluation, robot, machine learning

## Abstract

Rehabilitation training and movement evaluation after stroke have become a research hotspot as stroke has become a very common and harmful disease. However, traditional rehabilitation training and evaluation are mainly conducted under the guidance of rehabilitation doctors. The evaluation process is time-consuming and the evaluation results are greatly influenced by doctors. In this study, a desktop upper limb rehabilitation robot was designed and a quantitative evaluation system of upper limb motor function for stroke patients was proposed. The kinematics and dynamics data of stroke patients during active training were collected by sensors. Combined with the scores of patients’ upper limb motor function by rehabilitation doctors using the Wolf Motor Function Test (WMFT) scale, three different quantitative evaluation models of upper limb motor function based on Back Propagation Neural Network (BPNN), K-Nearest Neighbors (KNN), and Support Vector Regression (SVR) algorithms were established. To verify the effectiveness of the quantitative evaluation system, 10 healthy subjects and 21 stroke patients were recruited for experiments. The experimental results show that the BPNN model has the best evaluation performance among the three quantitative evaluation models. The scoring accuracy of the BPNN model reached up to 87.1%. Moreover, there was a significant correlation between the models′ scores and the doctors′ scores. The proposed system can help doctors to quantitatively evaluate the upper limb motor function of stroke patients and accurately master the rehabilitation progress of patients.

## 1. Introduction

Stroke is a disease caused by brain nerve injury. It is one of the common diseases among the elderly and the second leading cause of death [1]. Stroke patients usually have symptoms, such as hemiplegia, aphasia, and cognitive impairment, which may lead to death in severe cases [2]. Hemiplegia is the most common post-stroke symptom [3]. In most cases, the upper limbs of hemiplegic patients are more seriously affected than their lower limbs. Stroke rehabilitation is a process that enables patients to improve their limb motor function and return to normal life through rehabilitation training. Clinical experiments show that patients can recover limb motor function through rehabilitation training [4]. The recovery of motor function mainly occurs within the first three months after stroke [5]. Patients should participate in rehabilitation treatment as soon as possible and carry out rehabilitation training under the guidance of doctors.

The traditional rehabilitation methods mainly rely on the auxiliary training of rehabilitation doctors. The main task of rehabilitation doctors is to formulate rehabilitation plans to help patients to recover limb motor function [6]. In terms of rehabilitation evaluation, the traditional method is to use the evaluation scale, such as the Fugl-Meyer assessment (FMA) [7], Action Research Arm test (ARAT) [8], and modified Ashworth scale (MAS) [9] to evaluate the limb motor function of patients. The traditional rehabilitation evaluation method needs to be carried out by experienced rehabilitation doctors, which is a great waste of time and medical resources, and the evaluation results are greatly affected by the subjective influence of rehabilitation doctors. Compared with doctor-assisted rehabilitation training, the rehabilitation robot can provide a standardized training process and objectively record the trajectory, speed, force, and other data in the training process [10]. These data can be used to establish a quantitative evaluation model of patients’ motor function, help doctors to obtain patients’ rehabilitation progress more accurately and put forward targeted rehabilitation methods. Therefore, it is of great significance to establish a quantitative evaluation system of upper limb motor function of stroke patients, and help doctors adjust the rehabilitation training methods in time according to the evaluation results.

At present, many scholars have made attempts in rehabilitation evaluation. Lambercy et al. [11] introduced the results of a preliminary study using a virtual nail insertion test to evaluate the motor function of patients’ upper limbs. Lee et al. [12] proposed a virtual reality upper limb movement training system for stroke rehabilitation, which used a machine learning method to evaluate upper limb motor function. Miao et al. [13] designed a smartphone-based smart system to help stroke survivors improve upper limb rehabilitation. Patel et al. [14] received patients’ information through wearable sensors and used the random forest to evaluate patients’ motor function. Kim et al. [15] proposed a method to judge the severity of upper limb elbow spasm by using a machine learning algorithm. The main work of these scholars is to analyze the motor function of patients qualitatively and provide methods to classify the motor function of patients’ upper limbs based on motion data. Olesh et al. [16] developed a low-cost motion capture system to automatically evaluate upper limb injury in stroke patients. Liao et al. [17] proposed a framework based on deep learning to evaluate the quality of physical rehabilitation training automatically. Park et al. [18] proposed a modified Ashworth scale decision rule based on artificial intelligence and analyzed the effects of different biomechanical parameters on the MAS score. Cruz et al. [19] developed a portable motion capture system to obtain the three-dimensional kinematics data of upper limbs and then calculated the functional ability score through an automatic decision tree classifier. Otten et al. [20] used low-cost sensors to collect motion data and then obtain the patient’s upper limb function score through a machine learning algorithm. These scholars proposed a quantitative scoring method for the completion quality of a single action during rehabilitation training. Wang et al. [21] developed a multimodal fusion scheme to analyze the motor function of upper limbs comprehensively by using optical motion tracking equipment and wearable EMG equipment. The disadvantage of this method is that there are strict requirements for the bonding position of the electrode used for EMG monitoring. Kim et al. [22] developed an FMA evaluation tool using Kinect (Microsoft, Redmond, WA, USA) and verified it in stroke patients. Visual sensors, such as Kinect, require large experimental space and can only capture movements with large amplitude.

In this study, a desktop rehabilitation training robot with a simple structure and low cost was designed to collect the motion data and mechanical parameters of stroke patients in the process of rehabilitation training. Based on different machine learning algorithms, BPNN, KNN, and SVR, three rehabilitation evaluation models corresponding to the evaluation of the WMFT scale were established respectively. Finally, the performance differences of the three evaluation models were compared through experiments. Compared with the qualitative assessment methods proposed by Lambercy et al. and the single action quantitative assessment methods proposed by Olesh et al., our method can complete the quantitative assessment of the motor function of the entire upper extremity of stroke patients. We also avoid the disadvantages of visual sensors not being able to capture subtle movements and EMG devices requiring tight control over the placement of electrode pads. In addition, the rehabilitation robot proposed in this study is promising for application in home and community.

## 2. Materials and Methods

### 2.1. System Framework

**Figure 1:**
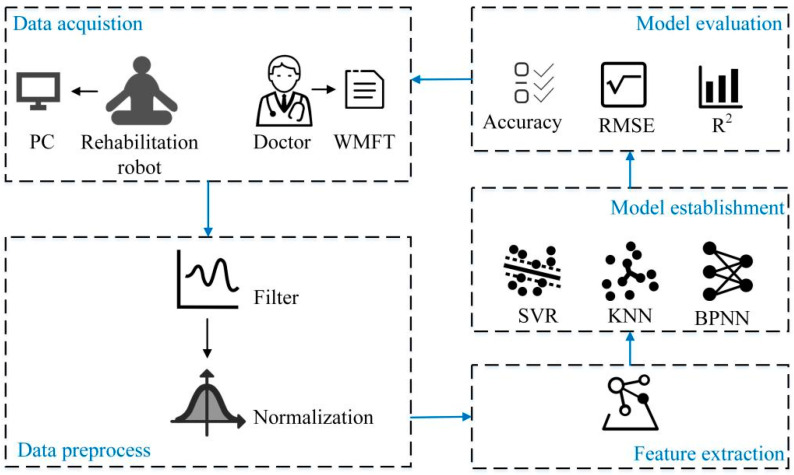
The quantitative evaluation system of upper limb motor function of stroke patients.

The proposed quantitative evaluation system of upper limb motor function of stroke patients is shown in Figure 1. In the data acquisition stage, a data acquisition system based on the rehabilitation robot is built to collect the kinematic and kinetic data of the subjects. The rehabilitation doctors used the WMFT scale to score the upper limb motor function of the subjects and the scores were used as labels to form a data set. The subjects′ data were then preprocessed; a moving average filter was used to process the data and the min-max method was used to normalize the data. Four features were extracted from the data, and three machine learning algorithms were used to establish the quantitative evaluation models of upper limb motor function. Finally, three indicators, including accuracy, root mean square error and determination coefficient, were selected to analyze and compare the scoring performance of these quantitative evaluation models.

**Figure 2:**
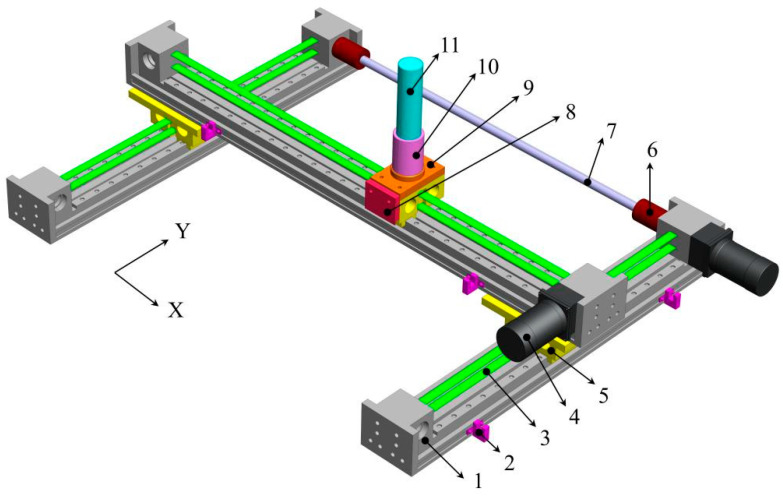
Upper limb rehabilitation robot model (Shell not shown). 1—slideway, 2—limit switch, 3—synchronous belt, 4—motor, 5—slider, 6—coupling, 7—transmission shaft, 8—plastic block, 9—base, 10—force sensor, 11—handle.

A desktop upper limb rehabilitation robot is developed to assist patients in active and passive training. The rehabilitation robot has a total length of 0.84 m, a width of 0.64 m, and a height of 0.35 m. The model of the upper limb rehabilitation robot is shown in Figure 2. Its mechanical structure is composed of synchronous belt modules, coupling, transmission shaft, limit switches, motors, sliders, base, a force sensor and handle. In passive training, two motors and synchronous belt modules drive the handle to drag the patient’s upper limbs to move on the plane. For safety reasons, photoelectric limit switches are installed at both ends of the synchronous belt modules. When the handle exceeds the specified position, the motor will power off automatically. In addition, an emergency stop switch is installed on the rehabilitation robot. During active training, the patient holds the handle and uses the upper limb to push the handle to move freely in the plane.

**Figure 3:**
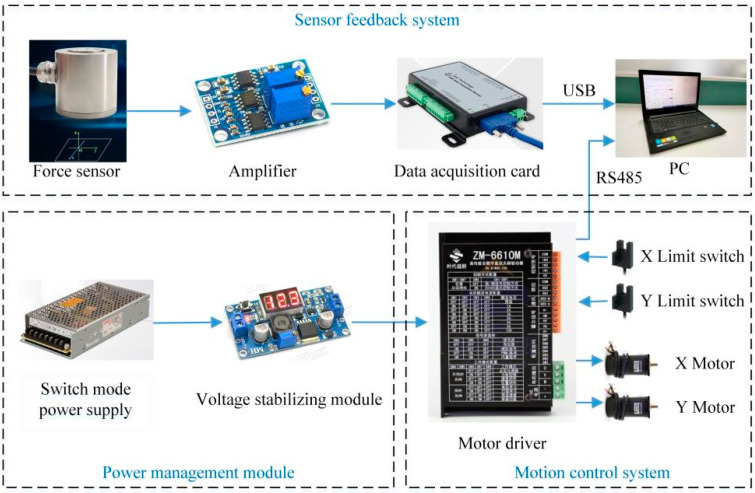
Hardware system block diagram.

The hardware system block diagram of the desktop upper limb rehabilitation robot is shown in Figure 3. The power management module is composed of a switch mode power supply (OMWO Co., Ningbo, China) with model S-145-24 and a voltage stabilizing module (Risym Co., Shenzhen, China) with model LM2596S. The 57BL95S06-210TF9 (Times brilliant Co., Beijing, China) brushless DC motors are adopted. The motor has a built-in Hall sensor, which can collect motor rotation angle and speed data. A ZM-6610M (Times brilliant Co., Beijing, China) motor driver is used to control the motors. EE-SX672-WR limit switches (Omron Co., Kyoto, Japan) are used to ensure that the handle moves within the safe range. An LZ-LW40 (Lizhi Co., Hefei, China) force sensor is used as the upper limb thrust sensing tool. The force sensor can simultaneously measure the forces in two mutually perpendicular directions, and the rated load of both directions is 100 N. The output signal of the force sensor is a voltage signal in the range of 0–10 mV. There is a linear relationship between input signal and output signal of the force sensor. To facilitate data acquisition, the AD620 amplifier was used to amplify the force sensor output signal by a factor of 300, so the voltage range after amplification is 0–3 V. USB3133A (ART Technology Co., Beijing, China) data acquisition card is used to collect upper limb thrust data, and the sampling frequency is set to 1000 Hz. The data acquisition card and motor driver use USB and RS485 communication methods to transmit data to the computer, respectively. MATLAB (MathWorks, Natick, MA, USA) is used for data processing, and LabVIEW (National Instruments, Austin, TX, USA) is used to write the control program.

### 2.2. Data Acquisition

#### 2.2.1. Experiment Design

**Figure 4:**
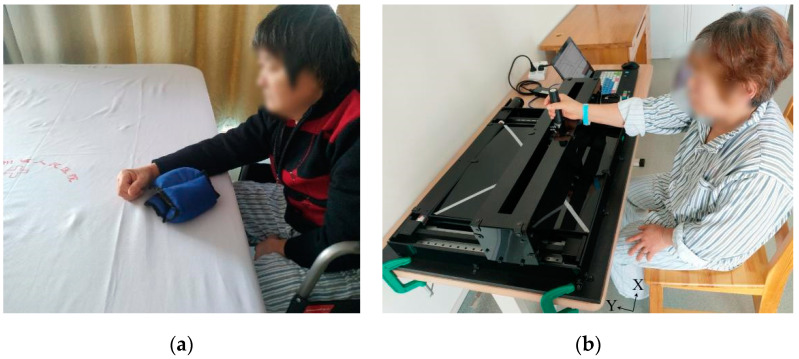
Scene of rehabilitation evaluation experiment. (**a**) WMFT evaluation scene. (**b**) Patients rehabilitation evaluation scene. (White lines are standard trajectories).

Subjects were recruited to use the upper limb rehabilitation robot for experiments. The upper limb motor function evaluation scenes are shown in Figure 4. As shown in Figure 4a, rehabilitation doctors were invited to evaluate the upper limb motor function of the subjects using the WMFT scale and Brunnstrom scale. The WMFT scale contains 15 tasks, with a full score of 5 for each task. A score of 0 indicates that the upper limb cannot complete the action, and a score of 5 indicates that the upper limb can complete the action normally. Brunnstrom staging scale divides the upper limb motor function into six stages [23]. Stage I indicates that the patient has no upper limb movement, and Stage VI indicates that the patient’s upper limb motor function is close to normal. When a doctor scores patients′ upper limb motor function alone, these scores will be affected by subjective factors. To reduce the impact of doctors’ subjectivity on the scoring results, five different doctors were invited to score the patients. The average scores of five doctors were taken as the final score. Before the experiment, rehabilitation doctors trained the subjects so that they could be familiar with the experimental process.

During the upper limb motor function evaluation experiment, the subject sat in front of the rehabilitation robot, as shown in Figure 4b. The experiment process is divided into three phases.

**Figure 5:**
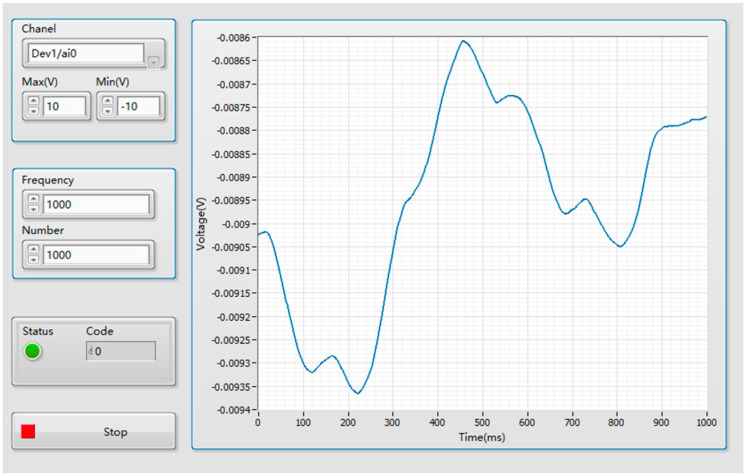
Data acquisition interface.

Phase1: Firstly, the subject holds the handle and moves it to one end of the *X*-axis. The subject then freely pushes the handle along the *X*-axis and the thrust information of the affected upper limb is collected through the force sensor. The data acquisition interface is shown in Figure 5.

Phase2: The subject pushes the handle freely along *X*-axis, and the speed data of the subjects’ upper limbs are recorded. The acceleration data are derived from the velocity data.

Phase3: The subject pushes the handle along the standard trajectory, and the actual motion trajectory of the handle is recorded.

#### 2.2.2. Participants

The rehabilitation evaluation experiment was conducted in Suzhou Xiangcheng people’s hospital. The experiment was approved by the ethics committee of Suzhou Xiangcheng people’s Hospital (Ethics committee approval No. 2018(006)) and the patients’ consent letters were obtained. The inclusion criteria of experimental subjects are as follows: (1) 18–70 years old; (2) first episode of stroke within three months before the experiment; (3) WMFT score 15–75, Brunstrom stage II or above; (4) good mental state and can communicate normally. The exclusion criteria are as follows: (1) more than one stroke (2) the patient has other obvious injuries in the upper limb, such as shoulder subluxation or fracture; (3) the patient has visual defects; (4) participating in other upper limb rehabilitation trials currently. A total of 10 healthy subjects (six males and four females) and 21 stroke patients with upper limb dysfunction (thirteen males and eight females) were recruited to participate in the experiment. The patients′ demographic information is shown in Table 1.

### 2.3. Data Preprocess

#### 2.3.1. Filter

**Figure 6:**
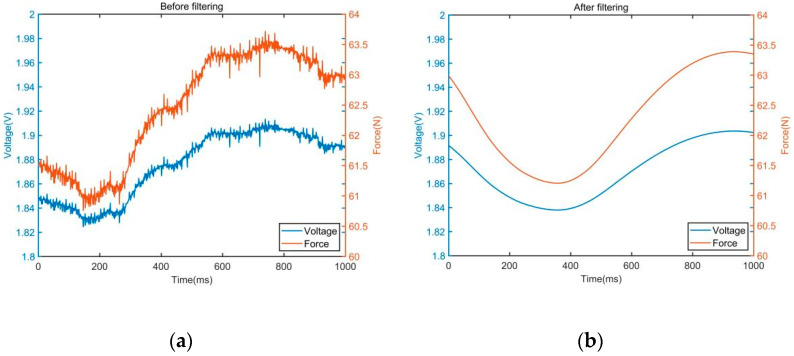
Signal comparison before and after filtering. (**a**) Data before filtering. (**b**) Data after filtering.

Due to the noise in the output signal of the force sensor, a moving average filter [24] is used to reduce the impact of noise on the experimental results. The expression of the moving average filter is shown in Equation (1).

(1) $$y_{\left(k\right)}=\frac{1}{N}\sum_{0}^{N-1}x_{\left(k-N\right)}$$

where $x_{\left(k\right)}$ is the original signal, $y_{\left(k\right)}$ is the filter output signal, and the filter window length *N* is 200. The comparison of data before and after filtering is shown in Figure 6. The blue curve represents the voltage signal collected by the data acquisition card, which varies from 0 to 3 V. The orange curve represents the subject’s upper limb thrust, which varies from 0 to 100 N.

#### 2.3.2. Normalization

To facilitate the subsequent establishment of a quantitative evaluation model based on machine learning algorithms and ensure that all values fall in the domain [0, 1], the Min-Max method [25] is used for normalization.

(2) $$x_{new}=\frac{x-x_{min}}{x_{max\;}-x_{min}}$$

where x is the original data and $x_{new}$ is the normalized data.$\;x_{min}$ is the minimum value of original data, and $x_{max}$ is the maximum value of original data.

### 2.4. Feature Extraction

**Figure 7:**
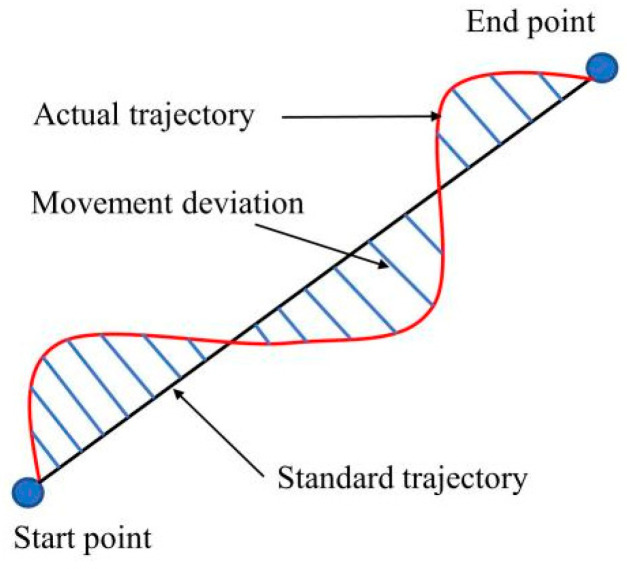
Schematic diagram of trajectory offset.

Lee et al. [12] have proved that there is a high correlation between the trajectory offset and average speed of upper limb movement in stroke patients and the results of WMFT score. Zhang et al. [26] mentioned that when doing the same upper limb movement, the speed and acceleration of stroke patients were significantly different from those of healthy subjects. Ekstrand et al. [27,28] pointed out that muscle weakness is the most common injury of upper limbs after stroke, which will reduce the ability of patients to use upper limbs in daily activities. Therefore, based on the above research results and the opinions of rehabilitation doctors, we selected the following four features:

(1) Trajectory offset *T*: the straight line between the start point and the end point is used as the standard trajectory to calculate the deviation between the actual trajectory of the patient’s hand movement and the standard trajectory. The schematic diagram of trajectory offset is showed in Figure 7, and the calculation formula of trajectory offset is

(3) $$T=\sqrt{\frac{\sum_{i=1}^{m}\Delta d_{i}{}^{2}}{m}}$$

where $\Delta d_{i}$ is the movement deviation, and *m* is the number of trajectory sampling points. The trajectory offset can be used to judge the jitter and muscle coordination ability of the patient’s upper limb.

(2) Average speed *V*: the average value of all instantaneous speeds. The calculation formula is shown in Equation (4).

(4) $$V=\frac{1}{N_{v}}(\sum_{i=1}^{N_{v}}V_{t})$$

where $V_{t}$ is the instantaneous velocity, and *N_v_* is the total number of seconds taken. The average speed reflects the continuity of strength in the process of rehabilitation training.

(3) Maximum instantaneous acceleration *a*: the maximum instantaneous acceleration of the patient’s upper limb. The calculation formula is shown in Equation (5).

(5) $$a=max\left(a_{t}=\frac{\Delta V_{t}}{\Delta t_{i}}\right)$$

where $a_{t}$ is the instantaneous acceleration, Δ*V_t_* is the instantaneous velocity variation, and Δ*t_i_* is the instantaneous time difference. The maximum acceleration can reflect the reaction speed of the patient’s upper limb during movement.

(4) Maximum thrust of upper limb *F*:

(6) $$F=max\left(F_{t}\right),t=1\cdot \cdot \cdot M$$

where *F_t_* is the sampling value and *M* is the total number of force sampling points. *F* reflects the muscle strength of the patients’ upper limbs.

### 2.5. Model Establishment

Using the scores of rehabilitation doctors as labels, the data of 31 subjects were composed into a data set. The 155 samples in the data set were randomly divided into a training set containing 124 samples and a test set containing 31 samples. Because BPNN, KNN, and SVR algorithms have a good nonlinear mapping ability and generalization ability [29], and the sample size of the dataset in this study is small, the above three algorithms are selected to establish the quantitative evaluation models of upper limb motor function.

#### 2.5.1. BPNN Model

**Figure 8:**
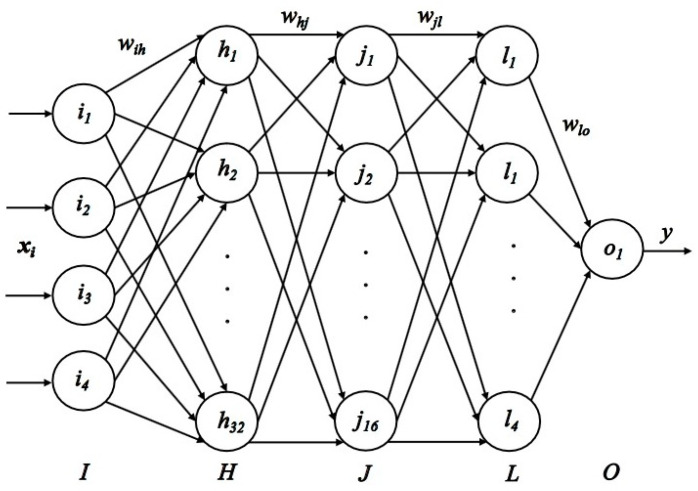
Structure diagram of BPNN.

BPNN is a multilayer feed-forward neural network with error backpropagation [30]. To obtain high accuracy and good generalization performance, BPNN is used to build the evaluation model. Figure 8 shows the established quantitative evaluation model of upper limb motor function based on BPNN. The neural network model has one input layer, three hidden layers, and one output layer. The input layer I has four neurons, the hidden layers H, J, and L have 32, 16, and 4 neurons respectively, and the output layer O has one neuron. The proposed four features are used as the input of the evaluation model, so the input can be written as $x_{i}=\left(F_{i},V_{i},a_{i},T_{i}\right)$, where i=1,2,⋯n. *y* is the output score of the neural network evaluation model. In the process of model training, the stochastic gradient descent is used as the optimization method, and the ReLU function is used as the activation function. The mean square error is used as the loss function, and the number of training steps is set to 1000.

#### 2.5.2. KNN Model

KNN regression algorithm aims to find the *k* nearest neighbors closest to the test points in the training set [31]. The training set can be written as $X_{i}=\left(F_{i},V_{i},a_{i},T_{i},y_{i}\right)$, where $y_{i}$ is the doctors’ scores. Note that a sample in the test set is ***X*** = (*F*,*V*,*a*,*T*,*y*), where *y* is the score of the KNN evaluation model. Traverse each point in the training set and calculate the Euclidean distance *Le* between it and sample ***X*** in the test set.

(7) $$L_{e}=\sqrt{{\left(F_{i}-F\right)}^{2}+{\left(V_{i}-V\right)}^{2}+{\left(a_{i}-a\right)}^{2}+{\left(T_{i}-T\right)}^{2}}$$

Sorting the obtained distance *Le* according to the size, and selecting *k* nearest neighbors ***X_j_*** (1 ≤ *j* ≤ *k*) in the training set. The average of the scores corresponding to these *k* nearest neighbors is used as the score of the KNN evaluation model. In this study, the number of nearest neighbors *k* is set to five, so

(8) $$y=\frac{1}{5}\overset{5}{\underset{j=1}{\sum }}y_{j}$$

#### 2.5.3. SVR Model

Forming the data of subjects into labeled training sets $S=\left\{\left(x_{1},y_{1}\right),\left(x_{2},y_{2}\right),\cdots ,\left(x_{n},y_{n}\right)\right\}$, where $x_{i}=\left(F_{i},V_{i},a_{i},T_{i}\right),x_{i}\in R^{n},y_{i}\in R,i=1,2,\cdots n$. The goal of SVR algorithm is to find a function *f(**x**)* and make it as close to $y_{i}$ as possible. Define

(9) $$f\left(x\right)=\omega^{T}x+b$$

where $\omega \in R^{n}$ is a high-dimensional weight vector, and the offset b∈R is a scalar. Note that a sample in the test set is X=(x,y), where x=(F,V,a,T). By applying Karush-Kuhn-Tucker theorem, Lagrange function is defined to solve SVR problem [32]. The score of the SVR model *y* can then be computed by using Equation (10).

(10) $$y=f\left(x\right)=\sum_{i=1}^{m}\left({\hat{\alpha }}_{i}-\alpha_{i}\right)K\left(x,x_{i}\right)+b$$

where $\alpha_{i}$, ${\hat{\alpha }}_{i}$ are Lagrange multiplier, $0\leq \alpha_{i},{\hat{\alpha }}_{i}\leq C$, $K\left(x_{i},x_{j}\right)=\phi x_{i}{}^{T}\phi x_{j}$ is a kernel function, and *ϕ* is a nonlinear operator, which maps the input data to a high-dimensional feature space. The kernel function used in the SVR evaluation model is RBF kernel, so

(11) $$K\left(x,x_{i}\right)=\exp\left(-\frac{{||x_{i}-x||}^{2}}{2\sigma^{2}}\right)$$

where *σ* is a free parameter of RBF kernel.

### 2.6. Model Evaluation Index

In this study, absolute error was used to reflect the error between the model’s score and the rehabilitation doctor’ score. The absolute error is defined as

(12) Absolute error=|model’s score−rehabilitation doctor’s score|

To compare the quantitative evaluation results of the above three regression algorithms (BPNN, KNN, and SVR) on the motor function of patients’ upper limbs, the accuracy, root mean square error (*RMSE*), and determination coefficient (*R*^2^) were used to evaluate the fitting performance of these models [33]. The definitions of accuracy, *RMSE*, and *R*^2^ are shown in Equations (13)–(15). In the experiment, the rehabilitation doctors thought that the error between the model′s score and the doctor′s score should be within 5. So, if the absolute error is less than 5, the score of the model is considered to be correct.

(13) $$Accuracy=\frac{N_{s}}{N_{t}}\times 100\%$$

(14) $$RMSE=\sqrt{\frac{1}{n}\sum_{i=1}^{n}{\left(y_{i}-\hat{y_{i}}\right)}^{2}}$$

(15) $$R^{2}=1-\frac{\sum_{i=1}^{n}{\left(y_{i}-\hat{y_{i}}\right)}^{2}}{\sum_{i=1}^{n}{\left(y_{i}-\bar{y_{i}}\right)}^{2}}$$

where $N_{s}$ refers to the number of samples whose absolute error is less than 5, and the total number of samples $N_{t}$ in the test set is 31. $y_{i}$ is the doctor’s score, $\bar{y_{i}}$ is the average value of $y_{i}$, and $\hat{y_{i}}$ is the model′s score.

## 3. Results

**Figure 9:**
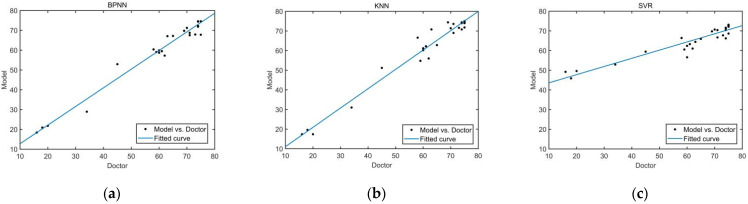
Comparison of scores of three evaluation models and rehabilitation doctors′ scores. (**a**) BPNN model. (**b**) KNN model. (**c**) SVR model.

Taking the four features of the trajectory offset, average speed, maximum instantaneous acceleration and maximum thrust of upper limb as the models’ inputs, the scores of each model can be calculated respectively. The scores calculated by the three quantitative evaluation models and the scores given by the rehabilitation doctors are shown in Figure 9.

**Figure 10:**
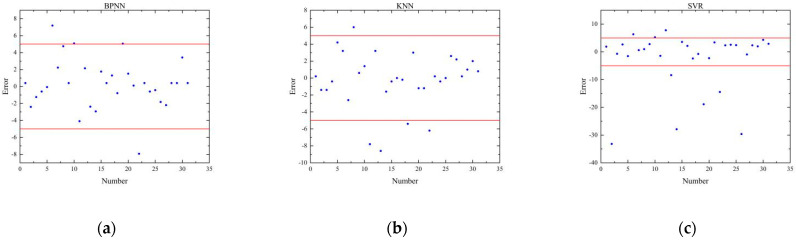
Scoring errors of three evaluation models. (**a**) BPNN model. (**b**) KNN model. (**c**) SVR model.

**Figure 11:**
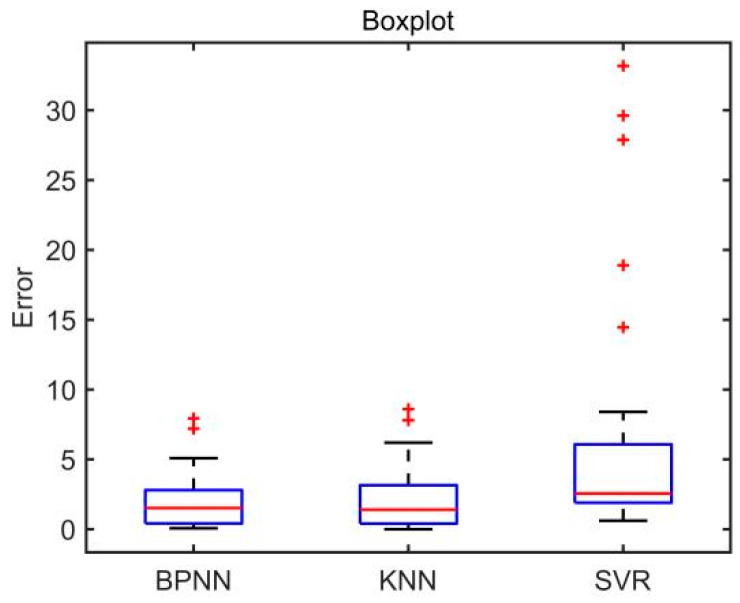
Boxplot of absolute error of the three models. Note: ‘+’ indicates an outlier.

The errors between the scores calculated by the three evaluation models and the scores given by rehabilitation doctors in the test set are shown in Figure 10. The boxplot of absolute errors of the three evaluation models is shown in Figure 11. As can be seen from Figure 11, the BPNN model and the KNN model have two outliers, while the SVR model has five outliers. It is calculated that the mean value of the absolute errors of the BPNN model is 2.09, which is smaller than that of the KNN model (2.24) and that of SVR model (6.14).

To verify the effectiveness of the evaluation models, the Pearson correlation test was used to analyze the correlation between the models′ scores and the rehabilitation doctors′ scores in the test set. SPSS (IBM Co., Armonk, NY, USA) was used for statistical analysis. The results show that the correlation coefficient (*R_c_*) between BPNN model′s scores and rehabilitation doctors′ scores is 0.986, which is bigger than that of the KNN model (0.983) and that of the SVR model (0.945). The *p*-values of the three evaluation models were all less than 0.05. Therefore, the scores calculated by the three models are significantly correlated with those given by rehabilitation doctors.

The accuracy, root mean square error (*RMSE*), determination coefficient (*R*^2^), correlation coefficient (*R_c_*) and absolute error (mean value ± standard deviation) of the three quantitative evaluation models are shown in Table 2. It can be concluded from Table 2 that the BPNN model has the best evaluation performance among the three quantitative evaluation models.

## 4. Discussion

In this study, a desktop upper limb rehabilitation robot was designed and a quantitative evaluation system of upper limb motor function for stroke patients was proposed. The kinematics and dynamics data of stroke patients during active training were collected by the desktop upper limb rehabilitation robot. Three different quantitative evaluation models based on BPNN, KNN, and SVR algorithms were established to evaluate the upper limb motor function of stroke patients, and experiments were carried out to compare the evaluation performance of these models.

The experimental results showed that the accuracy of the BPNN model, KNN model, and SVR model in scoring upper limb motor function were 87.1%, 83.87%, and 74.19%, respectively. The *RMSE* of the BPNN model, the KNN model, and the SVR model were 2.824, 3.222, and 10.838, respectively. The *R*^2^ of the BPNN model, the KNN model, and the SVR model were 0.973, 0.966, and 0.612, respectively. Compared with the KNN model and the SVR model, the BPNN model has higher accuracy, smaller *RMSE*, and smaller *R*^2^, indicating that the evaluation model based on BPNN has better evaluation performance.

Correlation analysis shows that the correlation coefficient between the BPNN model′s scores and doctors′ scores is 0.986, which is bigger than that of the KNN model (0.983) and that of the SVR model (0.945). Therefore, the experimental results show that the scores of the three evaluation models are all significantly correlated with the scores of doctors. The reason for the high correlation coefficient may be that the scores of rehabilitation doctors are used as the labels to train these models.

Limitations of our current study should also be noted. The total sample size of the data set used to establish the evaluation model is 155, and the relatively small number of samples in this study could limit the evaluation performance of the models. Therefore, more clinical trials are needed to collect more data to improve the accuracy of the evaluation model. There was an imbalance in the severity of the disease, as Brunnstrom stages of patients are mainly concentrated in stage II, V, and VI, but the number of patients in stage III and IV is small. Therefore, it is necessary to recruit more patients in stage III and IV in the future. In addition, this study only considers four features of upper limbs. More features will help to improve the performance of the evaluation model, and thus we need to explore the impact of other features on the performance of the evaluation model in future work.

## 5. Conclusions

In this study, a quantitative evaluation system was proposed to evaluate the upper limb motor function of stroke patients. Three different quantitative evaluation models based on BPNN, KNN, and SVR regression algorithms were established to evaluate upper limb motor function. Rehabilitation doctors were invited to conduct experiments on 10 healthy subjects and 21 stroke patients in combination with the WMFT scale. Compared with the scores of rehabilitation doctors, it was found that the accuracy of the established quantitative evaluation model reached up to 87.1%. The experimental results show that the BPNN model has the best evaluation performance among the three quantitative evaluation models. Moreover, there was a significant correlation between the models′ scores and the doctors′ scores. The proposed system will help doctors to evaluate the upper limb motor function of stroke survivors, and carry out individualized rehabilitation intervention for different patients. In the future, we will use more evaluation indicators and carry out large-scale clinical trials to improve the quantitative evaluation model. In addition, this study mainly studies the quantitative evaluation of upper limb motor function of stroke patients. In the future, experiments will be carried out to study the rehabilitation effect of the rehabilitation robot on patients.

## Figures and Tables

**Table 1:** Demographic information of patients with upper limb dyskinesia.

Subject	Age	Gender	Affected Side	Diagnosis (Day)	Brunstrom	WMFT
S1	63	Female	Left	12	V	63
S2	51	Male	Left	15	VI	70.2
S3	56	Female	Right	36	II	22.2
S4	51	Male	Left	8	VI	74.6
S5	63	Male	Left	22	II	16.4
S6	66	Female	Right	17	VI	71.4
S7	70	Female	Right	14	II	21.6
S8	48	Male	Right	10	VI	61.6
S9	57	Male	Right	6	V	57.6
S10	48	Male	Left	42	II	18.4
S11	61	Female	Right	14	II	25.4
S12	41	Male	Left	16	V	62.8
S13	50	Male	Left	9	IV	44.8
S14	56	Male	Right	13	VI	71.8
S15	63	Male	Left	68	III	33.8
S16	70	Male	Left	18	V	62.4
S17	56	Male	Right	20	VI	74.4
S18	69	Male	Left	14	VI	64.8
S19	62	Female	Left	75	II	22
S20	52	Female	Right	26	II	25.6
S21	18	Female	Right	7	VI	74.2

**Table 2:** Performance comparison of the three quantitative evaluation models.

Index	BPNN	KNN	SVR
Accuracy	87.1%	83.87%	74.19%
*RMSE*	2.824	3.222	10.838
*R* ^2^	0.973	0.966	0.612
*R_c_*	0.986	0.983	0.945
Absolute error	2.09 ± 2.08	2.24 ± 2.34	6.14 ± 8.88

## Data Availability

The data used in this study are available from the corresponding authors on reasonable request. The data are not publicly available because of participant confidentiality.
